# Primary and secondary infertility in Africa: systematic review with meta-analysis

**DOI:** 10.1186/s40738-020-00090-3

**Published:** 2020-12-02

**Authors:** Melese Shenkut Abebe, Mekbeb Afework, Yeshiwas Abaynew

**Affiliations:** 1 Department of Anatomy, School of Medicine, College of Medicine and Health Science, Wollo University, Dessie, Ethiopia; 2 Department of Anatomy, School of Medicine, College of Health Science, Addis Ababa University, Addis Ababa, Ethiopia; 3 Department of Biostatistics and epidemiology, School of Public Health, College of Medicine and Health Science, Wollo University, Dessie, Ethiopia

**Keywords:** Africa, Meta-analysis, Primary infertility, Proportion, Secondary infertility

## Abstract

**BACKGROUND:**

Infertility is a practical concern of Africans due to social disgrace and exclusion. This meta-analysis aims to analyze the proportion of primary and secondary infertility and identify the etiologic factors based on the studies conducted in Africa.

**METHODS:**

An internet-based search was conducted on the following databases; PubMed/Medline, EMBASE, Cochrane library, and google scholar. Both population and institution-based studies conducted among African couples, males, and females were included. Data extraction and critical appraisal of the articles were done by two independent investigators. Meta-analysis using a random effect model was conducted by Stata version 14. Forest plot, heterogeneity test, and funnel plot for publication bias were performed.

**RESULTS:**

The pooled proportion of primary and secondary infertility in Africa was 49.91% (I^2^ = 98.7, chi-square = 1509.01, degree of freedom = 19 and *p* < 0.001) and 49.79% (I^2^ = 98.7, chi-square = 1472.69, degree of freedom = 19 and *p* < 0.001) respectively. The pooled prevalence of the causes of infertility indicated that 54.01% and 22.26% of the infertility cases were respectively due to female and male-related problems. In 21.36% of infertility cases, both sexes were affected, while 10.4% of the causes of infertility were unexplained. The pooled prevalence of mostly reported causes of male infertility was 31% (oligospermia), 19.39% (asthenozoospermia), and 19.2% (varicocele). The most commonly identified causes of female infertility were pelvic inflammatory disease, tubal factors, and abortion with a pooled prevalence of 39.38%, 39.17%, and 36.41% respectively.

**Conclusions:**

In Africa, the proportion of primary and secondary infertility is approximately equal. Infertility is mostly due to female-related causes like; pelvic inflammatory diseases, uterine tube related problems, and abortion. Oligospermia, asthenozoospermia, and varicocele were the commonest causes of male-related infertility. It is suggested that interpretation and utilization of these findings should consider the presence of substantial heterogeneity between the included studies.

## Introduction

The clinical definition of infertility is an inability to be pregnant after 12 months or more of regular unprotected coitus [1]. From the demographer’s point of view, infertility is defined as the absence of live birth in a woman of reproductive age (15–49 years) with regular unprotected sexual intercourse [2]. Infertility is classified as primary or secondary. Primary infertility is denoted for those women who have not been conceived previously. In secondary infertility, there is at least one conception but fails to repeat [2].

The etiologic sources of infertility can be of either the man or the woman or both. In developing countries, most of the causes are attributed to infection. The majority of African women infertility is due to infectious causes [3] and about 46% of Sub-Sahara African men have infertility related to sexually transmitted diseases [4].

For women, bilateral uterine tube blockage is the commonest cause of infertility [5]. The fallopian tubal occlusion is mainly due to pelvic inflammatory disease (PID) which is caused by post-abortal and post-partum infections [6]. In addition, ovulatory disorder, contraception use, and sociocultural factors are the causes of female infertility. Prolonged use of oral contraception, cultural factors like feeding habit, and male heat exposure are reported risk factors affecting fertility [7, 8]. Genetic and environmental factors and infections can affect male fertility. These could lead to impaired sperm cell production, sperm transportation, and sexual habit which end up with infertility [6]. Furthermore, the etiology of infertility shows a significant regional variation [9].

In many African countries, the success of marriage overlies on the ability of a woman to bear children. Being infertile results in a serious psychological trauma and social stigma. In some cases, it may end up with social disgrace and exclusion, verbal and physical abuse, and marriage violence and breakup. Especially for women, infertility significantly reduces their quality of life, expose for multiple sexual partners, sexually transmitted diseases, increased sexual dysfunction, and poor kinship [10]. Therefore, it is a real personal, social, and public health issue, mainly in developing countries.

Although infertility is a global issue, the majority of its causes are reported from the third world nations. It is a practical concern for Africans due to the high social stigma [4]. The magnitude of infertility is reported worldwide differently. The infertility rate ranges from 5–30% as reported for different countries [11].

Regardless of the widespread consequences of infertility, the provision of infertility medical care is limited in developing countries including Africa [12]. To design appropriate treatment modalities, the pooled estimation of infertility proportion and etiologic factors plays a central role. Thus, the review question for this meta-analysis is: what is the pooled proportion of primary and secondary infertility and its etiologic factors in Africa?

## METHODS

### Search strategy

For a purpose of identifying the pooled proportion of primary and secondary infertility and its etiologic factors in Africa, a comprehensive internet-based search was done on the following databases; PubMed/Medline, EMBASE, Cochrane library, and google scholar. The following MESH terms were used; Infertility OR sub-fertility OR sterility OR subfecundity OR infecundity OR subfertility OR childlessness AND prevalence OR incidence OR epidemiology OR proportion AND “risk factors” OR “associated factors” OR determinants OR etiology. Additionally, the references of retrieved studies were examined to identify further articles. In addition, a manual search for a thesis/dissertation was performed on University websites. The last date of the search was August 1, 2019. The search was limited to articles published in the English language. Specifically, the search detail on PubMed (first searched database) was; (((((Infertility OR sub-fertility OR sterility OR subfecundity OR infecundity OR subfertility OR childlessness))) AND ((prevalence OR incidence OR epidemiology OR proportion))) AND ((“risk factors” OR “associated factors” OR determinants OR etiology))) AND (name of the country AND English[lang]). This review and meta-analysis were guided by Preferred Reporting Items for Systematic Reviews (PRISMA) guidelines 2009 [13].

### Eligibility criteria

#### Inclusion criteria

Studies that reported the proportion of primary/secondary infertility and related factors/etiology were included.

### Study area and design

All observational studies (cross-sectional, case series, cohort, and case-control) that reported primary data on the proportion of primary/secondary infertility, related factors, and conducted in Africa were considered in this study.

### Publication year and language

Both published and unpublished (thesis/dissertation) articles written in English and found until August 1, 2019 were incorporated.

### Population

Studies conducted among couples, males, or females were taken into account.

### Exclusion criteria

Articles were excluded if only abstract is accessed and full-text request from the author was not possible within two weeks. The email request was done by MS.

### Data extraction

Data extraction was performed by an excel spreadsheet. Information regarding the author’s name, publication year, name of the country in which the study was conducted, type of study design, total sample size, total infertility, the proportion of primary and secondary infertility, and etiology/risk factors were included in the data extraction sheet.

### **Outcome measurement**

This systematic review has two outcomes. The first was the proportion of primary and secondary infertility among the total infertility. This was done by dividing the number of cases with primary/secondary infertility to the total infertility and multiplying by 100. The second outcome was to calculate the prevalence of etiology/risk factors of infertility. The classification of etiology/risk factors was conducted based on outcome reports in the included studies. Each etiology/risk factor was analyzed individually if they were mention at least by two studies. The overall prevalence of commonly reported etiologies/risk factors was calculated by dividing the number of infertility due to each etiologies/risk factors by the total number of infertilities multiplied by 100.

### Quality assessment

The quality of the included articles was rigorously assessed by MS and YA independently. For quality assessment, Newcastle-Ottawa quality assessment scale modified for cross-sectional [14], cohort [15], and case-control/series studies [16] was used. The scale has three segments. The first section with a maximum of five stars assesses the methodological quality of studies. In this section, the lowest score indicates poor quality while the highest score is for good quality studies. The second and third parts of the tool determine the comparability, statistical analysis, and outcome report of each study. They have two and three maximum stars respectively. Overall, articles that scored 50% and above of the quality assessment were included in the meta-analysis [15]. Difference between investigators (data extractor and quality assessor) in the score and decision on the quality of the papers were resolved by discussion.

### Statistical analysis

Data analysis was performed by Stata version 14 software. Meta-analysis was performed to estimate the pooled proportion of primary and secondary infertility from total infertility and the pooled prevalence of etiologic factors accompanied by the 95% confidence intervals. Between study heterogeneity was assessed using I^2^ and Cochran’s Q method. The value of I^2^ greater than 75% was considered as high heterogeneity [17]. Due to the presence of heterogeneity, a random-effect meta-analysis was conducted. In addition, subgroup analysis was performed by the four regions of Africa and year of studies. Egger test was conducted and a funnel plot was drawn to check for the presence of publication bias. For heterogeneity and Egger tests, a p-value less than 0.05 was considered as statistically significant.

## RESULTS

### Search results

**Fig. 1:**
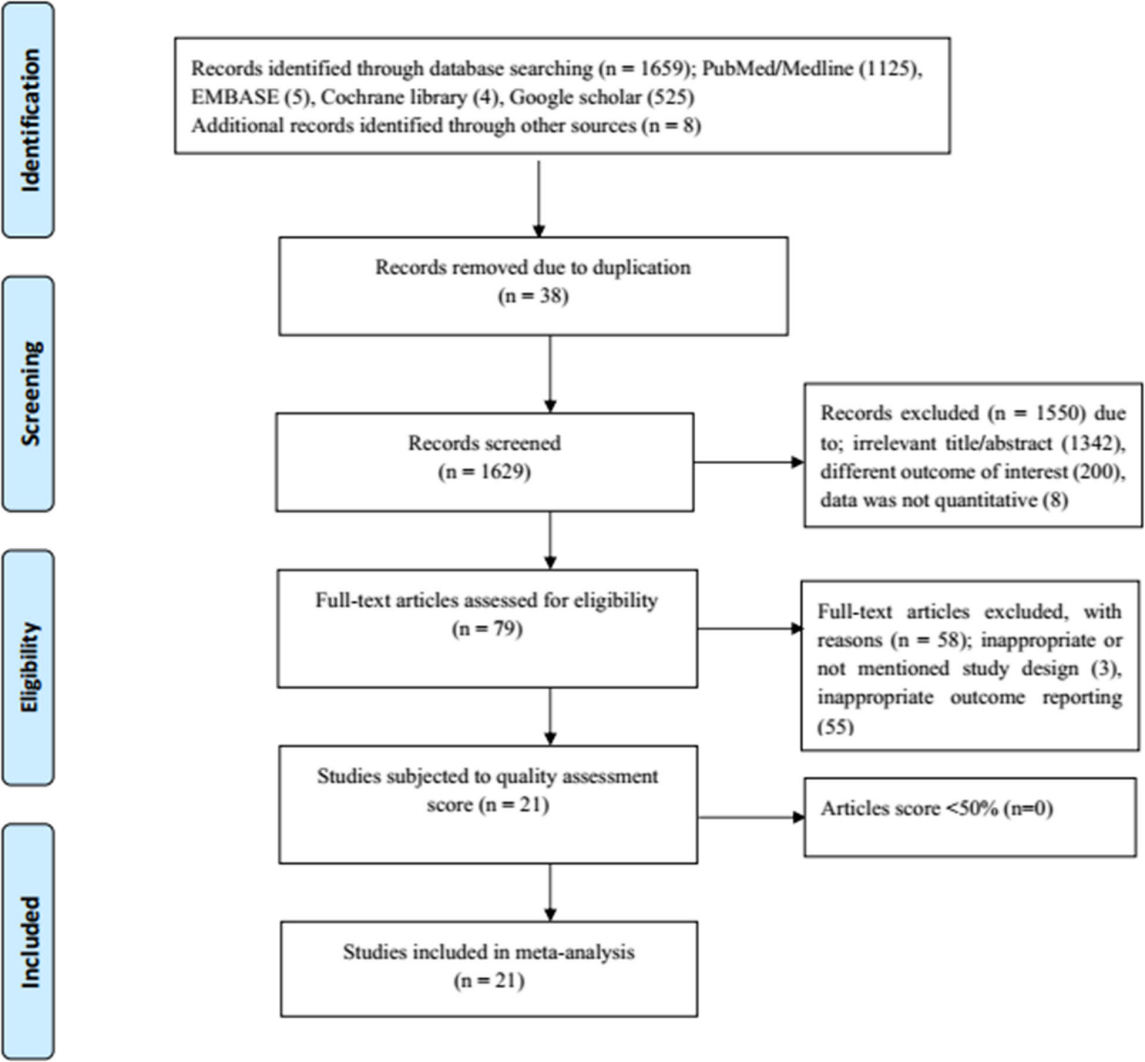
Prisma flow diagram showing screening and selection of studies for the systematic review and meta-analysis

A total of 1659 articles were obtained during the initial search on PubMed/Medline, EMBASE, Cochrane library, and Google Scholar. Additional eight articles were collected through manual searches from University websites and article references. One thousand six hundred forty-six articles were excluded due to duplication, irrelevant title/abstract, the reported outcomes were not our interest or qualitative and not inappropriate study design. In addition, 21 full-text articles were subjected to quality assessment. Fortunately, all 21 articles scored above 50% in the Newcastle-Ottawa quality assessment scale. These articles reported the proportion of primary and secondary infertility and/or the prevalence of the etiology of infertility. The detailed process of articles selection is presented in Fig. 1. The summary of main details of the included studies was presented in Table 1.

### Pooled proportion of primary infertility in Africa

**Fig. 2:**
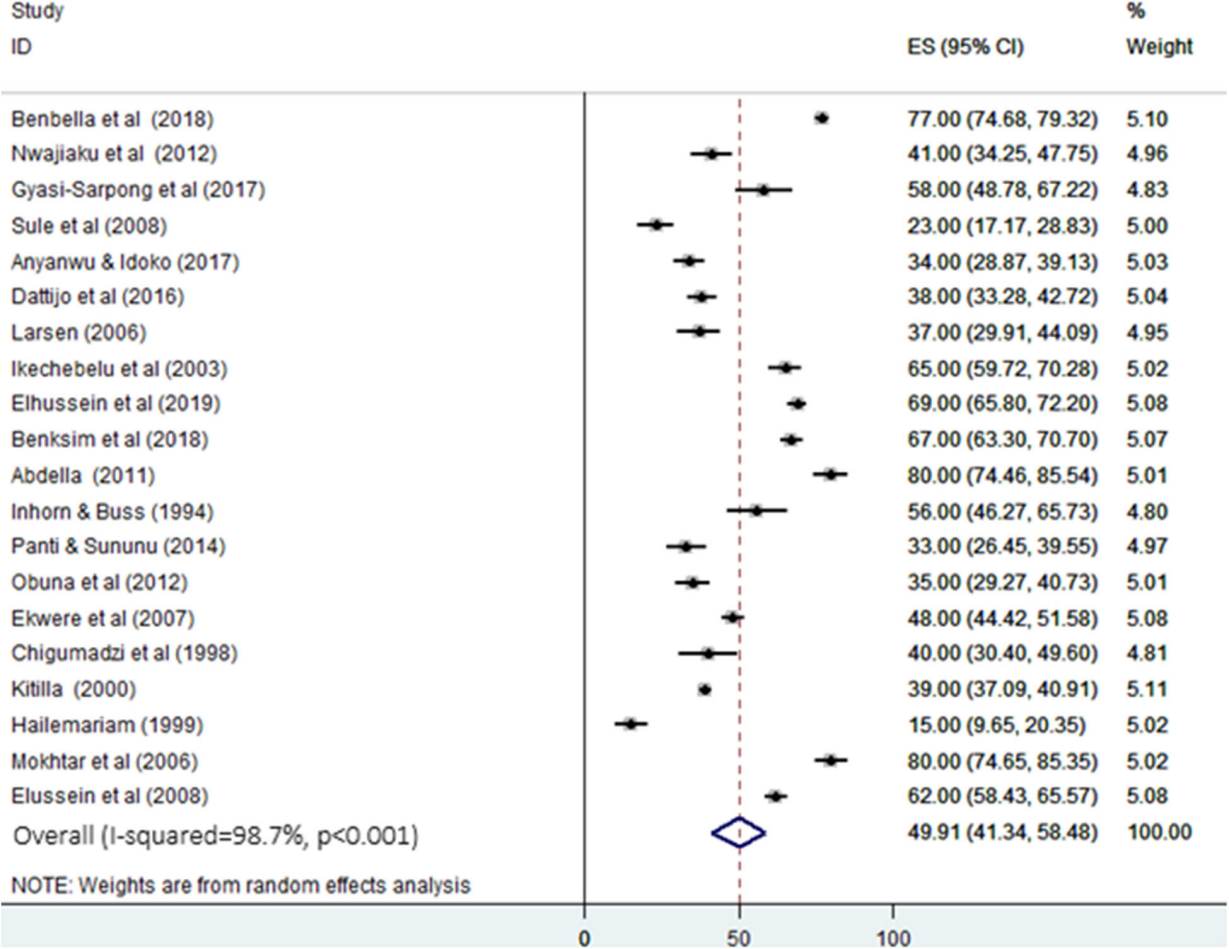
Forest plot for the pooled proportion of primary infertility in Africa. ES: effect size (%) & CI: confidence interval

The result of this meta-analysis (random effect model) indicated the overall pooled proportion of primary infertility in Africa was 49.91% (95% CI; 41.34 to 58.48). This analysis was based on 20 studies. The variability between studies was substantially high (heterogeneity I^2^ = 98.7, heterogeneity chi-square = 1509.01, degree of freedom = 19, and *p* < 0.001) (Fig. 2). The heterogeneity of the literatures can be due to unrepresentative sample size, the difference in population, year of study, and etiology of infertility.

**Table 1:** Description of the included studies

No	Name of Authors	Year of Publication	Study done	Type of study	SS (n)	PI (%)	SI (%)	MF (n)	FF (n)	CF (n)	UF (n)	Study region
1	Benbella et al. [18]	2018	Morocco	Case series	1265	77	23	NR	NR	NR	NR	North Africa
2	Nwajiaku et al. [19]	2012	Nigeria	Cross-sectional	204	41	59	25	45	20	10	West Africa
3	Gyasi-Sarpong et al. [20]	2017	Ghana	Cross-sectional	110	58	42	15	85	NR	NR	West Africa
4	Sule et al. [21]	2008	Nigeria	Case study	200	23	78	6.5	93.5	NR	NR	West Africa
5	Anyanwu & Idoko [22]	2017	Gambia	Cross-sectional	328	34	59	9	NR	NR	10	West Africa
6	Dattijo et al. [23]	2016	Nigeria	Cross-sectional	406	38	62	11	67	10	12	West Africa
7	Larsen [24]	2006	Tanzania	Cross-sectional	178^a^	37	63	9	66	13	12	East Africa
8	Ikechebelu et al. [25]	2003	Nigeria	Cross-sectional	314	65	35	42	26	21	11	West Africa
9	Elhussein et al. [26]	2019	Sudan	Cross-sectional	800	69	31	36	43	18	3	North Africa
10	Benksim et al. [27]	2018	Morocco	Cross-sectional	619	67	33	NR	NR	NR	NR	North Africa
11	Abdella [28]	2011	Sudan	Cohort	200	80	21	20	38	31	11	North Africa
12	Inhorn & Buss [8]	1994	Ejypt	Case control	100^b^	56	44	46	82	NR	6	North Africa
13	Panti & Sununu [29]	2014	Nigeria	Cross-sectional	198	33	67	20	43	17	21	West Africa
14	Obuna et al. [30]	2012	Nigeria	Cross-sectional	266	35	65	23	35	24	18	West Africa
15	Ekwere et al. [31]	2007	Nigeria	Cross-sectional	750	48	51	30	58	12	NR	West Africa
16	Chigumadzi et al. [5]	1998	South Africa	Cross-sectional	100	40	60	NR	NR	NR	3	Southern Africa
17	Kitilla [32]	2000	Ethiopia	Cross-sectional	2503	39	61	NR	NR	NR	NR	East Africa
18	Hailemariam [33]	1999	Ethiopia	Cross-sectional	171	15	85	NR	NR	NR	NR	East Africa
19	Mokhtar et al. [34]	2006	Ejypt	Case control	215	80	20	NR	NR	NR	NR	North Africa
20	Eric et al. [35]	2016	Burkina Faso	Cross-sectional	93	NR	NR	35	43	22	NR	West Africa
21	Dhont et al. [36]	2011	Rwanda	Cohort	224	NR	NR	NR	31	50	3	East Africa

*SS* Sample size, *PI* Primary infertility, *SI* Secondary infertility, *MF* Male factor, *FM* Female factor, *CF* Combined factor, *UF* Unidentified factor.

^a^only 91 of 178 infertility cases were investigated to identify the factors

^b^MF was calculated from 87 of 100 cases.

### Subgroup analysis of primary infertility by regions and year of studies

Subgroup analysis was conducted to estimate the regional difference in the proportion of primary infertility due to the presence of heterogeneity. The selected studies were conducted in four regions of Africa. Included West African countries were; Burkina Faso, Ghana, Gambia, Niger, and Nigeria. North African countries were Egypt, Morocco, and Sudan. Ethiopia, Rwanda, and Tanzania were included countries in the East Africa region. Only one research from South Africa met the inclusion criteria in the Southern region of Africa.

**Fig. 3:**
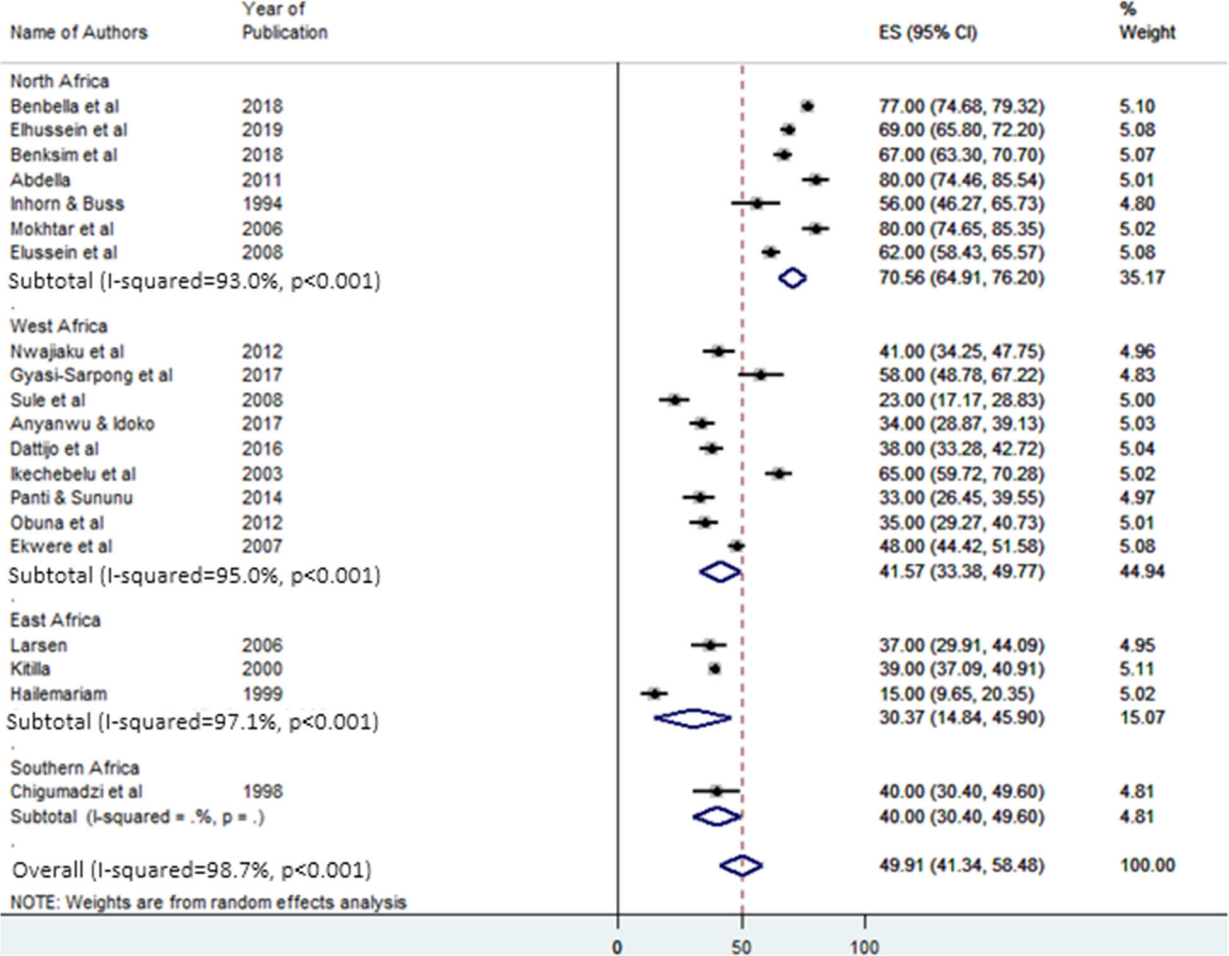
Forest plot of subgroup analysis for the pooled proportion of primary infertility in Africa. ES: effect size (%) & CI: confidence interval

The highest pooled proportion of primary infertility was reported from North Africa. It was 70.56% (95% CI: 64.91–76.2%). The lowest was from East Africa, 30.37% (95% CI: 14.84–45.9%). The pooled proportion of primary infertility in West Africa was 41.57% (95% CI: 33.38–49.77%). In Southern Africa, the result was from only one study and it was 40% (95% CI: 30.4–49.6%) (Fig. 3).

Additionally, subgroup analysis by the year of studies was conducted to check whether the year of the studies affected the pooled proportion of primary/secondary infertility. In this analysis, the results showed that the studies conducted before 2000 reported a lower (36.75%) pooled proportion of primary infertility. On the other hand, the pooled proportion of primary infertility was higher (57.26%) in studies conducted after 2015 (Table 2).

**Table 2:** Subgroup analysis of primary/secondary infertility by year of studies

		Number of studies	Pooled proportion (95% CI)	Heterogeneity (I^2^, *p*-value)
Year of studies (PI)	Before 2000	3	36.75 (11.08–62.45)	96.7%, *p* < 0.001
	2000–2004	2	51.88 (26.4-77.36)	98.8%, *p* < 0.001
	2005–2009	5	50.08 (33.34–66.83)	98.4%, *p* < 0.001
	2010–2014	4	47.28 (24.2-70.36)	98.2%, *p* < 0.001
	2015–2019	6	57.26 (43.45–71.06)	98.7%, *p* < 0.001
Year of studies (SI)	Before 2000	3	63.25 (37.55–88.94)	96.7%, *p* < 0.001
	2000–2004	2	48.12 (22.64–73.6)	98.8%, *p* < 0.001
	2005–2009	5	49.92 (32.96–66.88)	98.5%, *p* < 0.001
	2010–2014	4	52.97 (30.58–75.36)	98.1%, *p* < 0.001
	2015–2019	6	41.55 (28.92–54.19)	98.4%, *p* < 0.001

*PI* primary infertility, *SI* secondary infertility, *CI* confidence interval

### Pooled proportion and subgroup analysis of secondary infertility in Africa

**Fig. 4:**
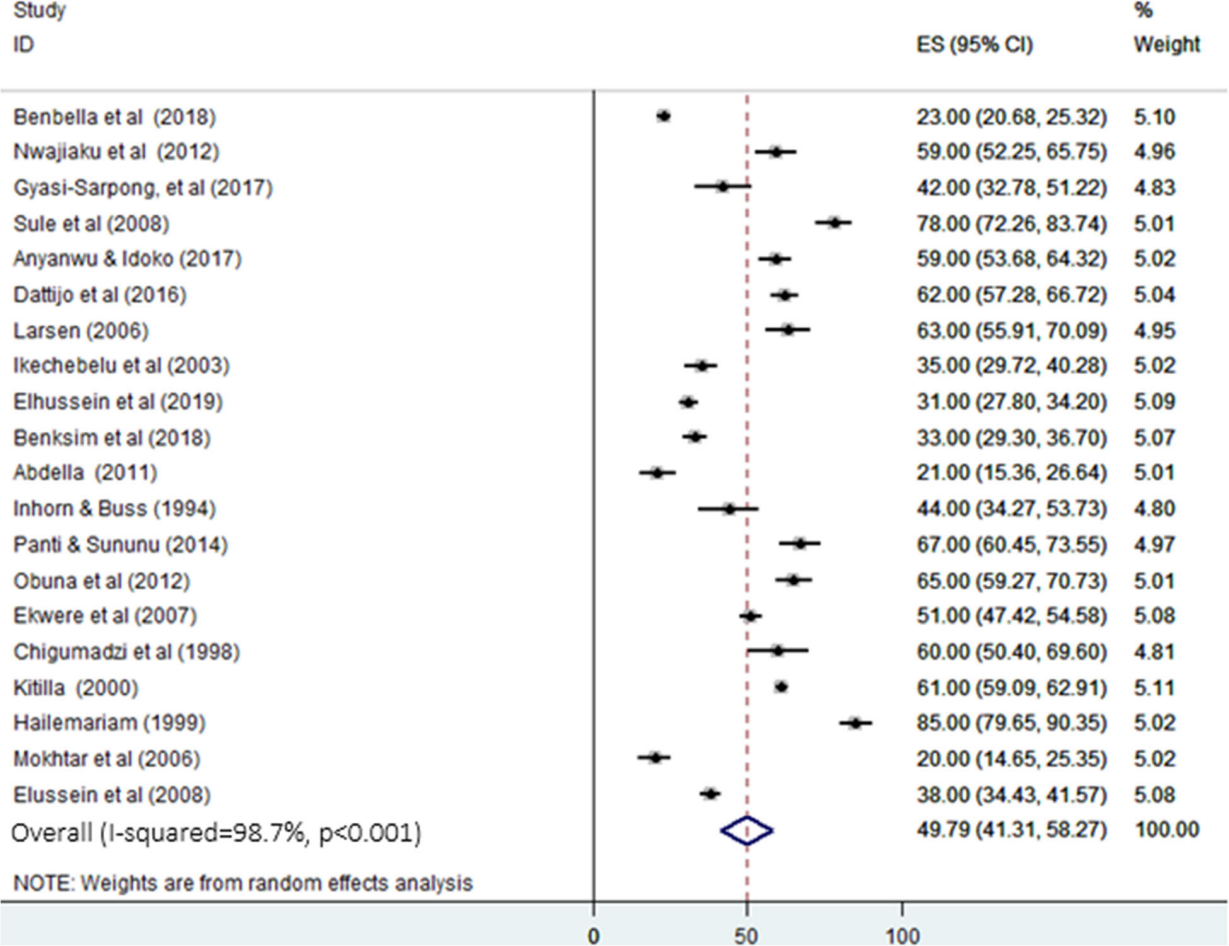
Forest plot for the pooled proportion of secondary infertility in Africa. ES: effect size (%) & CI: confidence interval

**Fig. 5:**
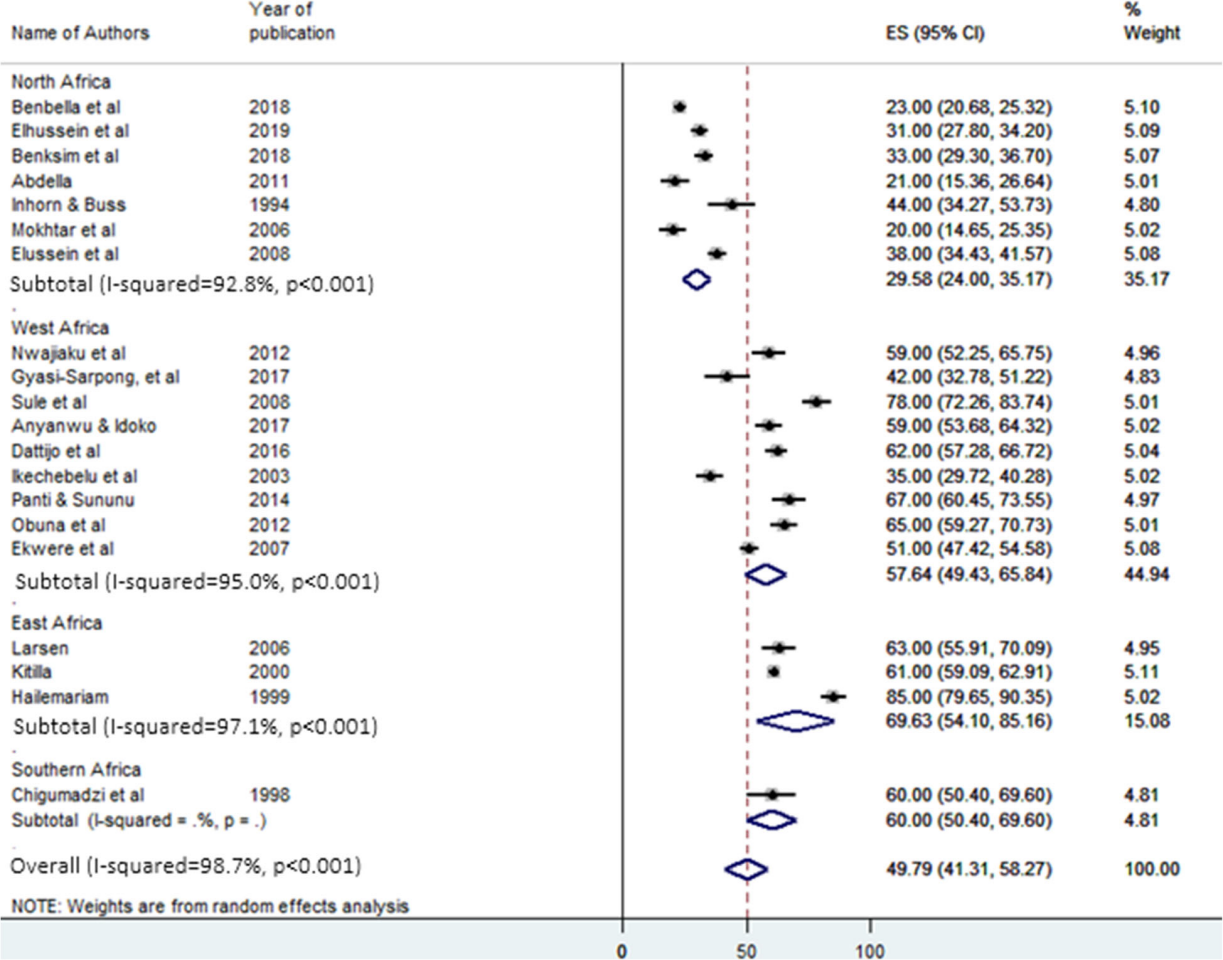
Forest plot of subgroup analysis for the pooled proportion of secondary infertility in Africa. ES: effect size (%) & CI: confidence interval

As presented in Fig. 4, the pooled proportion of secondary infertility from the total infertility cases was 49.79% (95% CI: 41.31%-58.27%) with heterogeneity I^2^ = 98.7, heterogeneity chi-square = 1472.69, degree of freedom = 19 and *p* < 0.001. In the subgroup analysis for secondary infertility, in contrast to primary infertility, the highest and the lowest pooled proportion was observed in East Africa (69.63%, 95% CI: 54.1%-85.16%) and North Africa (29.58%, 95% CI: 224%-35.17%) respectively (Fig. 5). Additionally, the pooled proportion of secondary infertility was 63.25% and 41.55% in studies conducted before 2000 and after 2015, respectively (Table 2).

### Male and female contribution to infertility

Sources of infertility were calculated from 14 studies reported male and female cause and 11 and 12 studies reported combined and unexplained causes of infertility, respectively. The pooled estimation of male and female-related causes of infertility was analyzed from studies conducted in seven African countries. Female’s contribution to infertility accounts for about 54.01% (95% CI: 41.49%-66.52%) of the cases. The studies were substantially heterogeneous; heterogeneity chi-square = 1031.23, degree of freedom:13, I^2^ = 98.7%. In spite of this, male factors contributed to 22.26% (pooled estimate) (95% CI: 16.5%-28.74%) of infertility. However, the pooled estimate of both sex contribution to infertility was also 21.36% (95% CI: 16.06%-26.66%). On the other hand, some causes of infertility were not explained/identified. The pooled estimation for these unexplained causes of infertility was 10.4% (95% CI: 6.89%-13.92%) (Figures are not shown here).

### Commonly reported causes of infertility

As shown in Table 3, the pooled prevalence for each cause of infertility was conducted separately. Their pooled estimate indicated that the commonest reported causes of male infertility were oligospermia (31%), asthenozoospermia (19.39%), and varicocele (19.2%). The pooled prevalence of the commonest causes of female infertility was 39.38%, 39.17%, and 36.41% for pelvic inflammatory disease (PID), tubal factors, and abortion respectively.

### Assessment of publication bias and small study effect

**Fig. 6:**
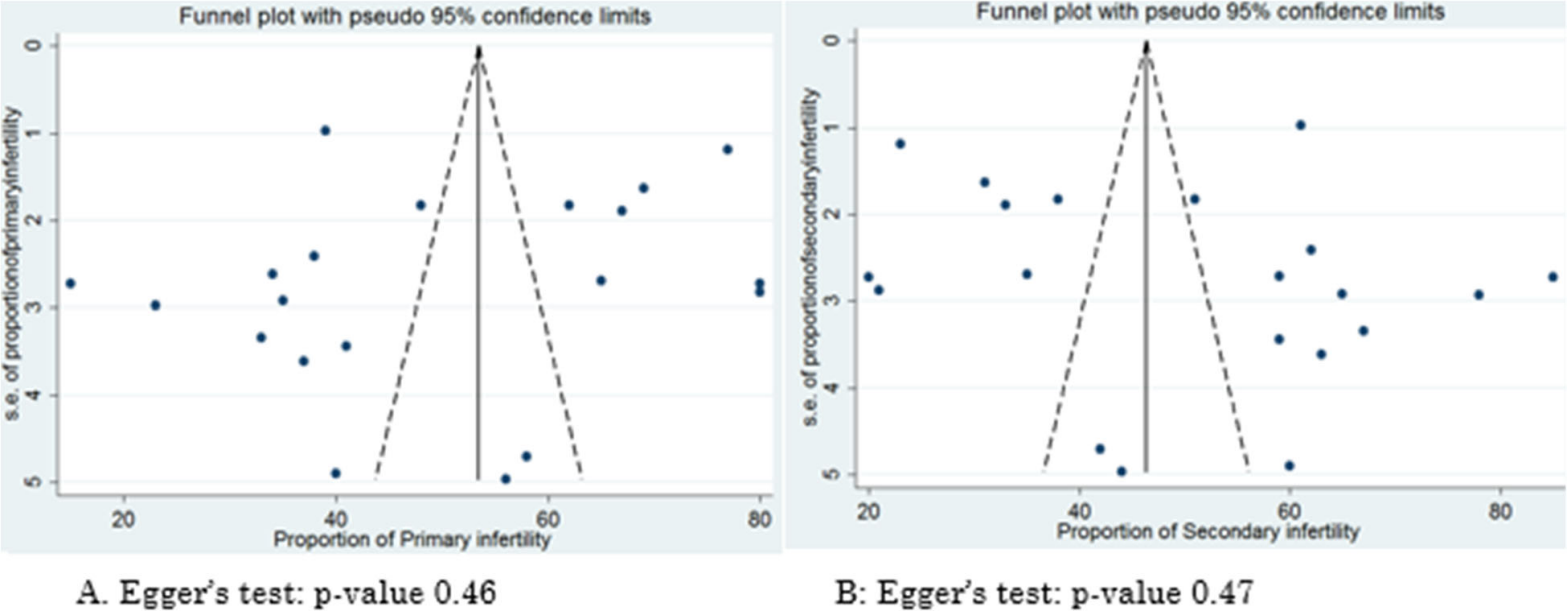
Funnel plot for assessing publication bias in the proportion of primary and secondary infertility

Egger’s test and visual inspection of the funnel plot (Fig. 6) were performed to assess the presence of publication bias. The result indicated there was no publication bias with *p*-value > 0.05.

**Table 3:** Summary table of the data from included studies showing the pooled prevalence of the causes of male and female infertility using the random effect model

Causes of male infertility^a^	Number of studies/SS	Pooled prevalence %	Causes of female infertility	Number of studies /SS	Pooled prevalence %
Oligospermia	10 (2486)	31	Tubal factor	12 (2767)	39.17
Asthenozoospermia	7 (1522)	19.39	Ovulatory disfunction	10 (2528)	31.47
Azoospermia	10 (2486)	14.24	Uterine factor	12 (2886)	18.55
Varicoceles	6 (2435)	19.12	PID	5 (1136)	39.83
Cryptorchidism	4 (1506)	8.9	Endometriosis	5 (1041)	1.65
Teratozoospermia	4 (1435)	7.77	Abortion	3 (502)	36.41
Oligo-asthenozoospermia	3 (1285)	15.12	Cervical factor	3 (395)	27.45
			Puerperal sepsis	3 (502)	19.14

*PID* pelvic inflammatory disease, *SS* sample size

^a^For the causes of male infertility, the aforementioned terms in the table are based on the following definitions. Oligospermia is a condition when the number of spermatozoa is less than 15 million per a milliliter of semen. Azoospermia is a name given for the total absence of sperm cells in semen. When progressively motile sperm cells are less than 32% or total motility is less than 40%, it is named as asthenozoospermia. If the number of a sperm cell with abnormal morphology is greater than 4%, it is teratozoospermia. In case when oligospermia and asthenozoospermia found simultaneously the name will be oligo-asthenozoospermia

## Discussion

Infertility is a worldwide public health agenda affecting the personal, social, and economic life of an individual and the family as a whole. The difference in terms of definition, diagnostic cut points, study design, and source population make performing a meta-analysis on infertility difficult. The prevalence, classification, and causes of infertility are reported in population-based studies, demographic and health survey report or institution-based studies. Each method has its own advantages and disadvantages. The present meta-analysis used both population and institution-based studies which analyzed the proportion of primary and secondary infertility from the total infertility and report the prevalence of its causes. It analyzed the proportion of primary and secondary infertility and summarize the etiology of infertility.

In this meta-analysis, the included studies have reported a heterogeneous proportion of primary and secondary infertility. This difference in the proportion of infertility was depended on the reported causes of infertility. For instance, in study areas where sexually transmitted infections (STI) and infection following the first pregnancy or abortion are common, the proportion of secondary infertility was higher [29, 32, 33]. Whereas in areas where the management of the above conditions is relatively good, primary infertility outnumbers secondary infertility [18, 27, 34] (Table 1). However, the overall pooled proportion of primary and secondary infertility at the level of Africa was almost similar. This finding is in difference from a meta-analysis result in Iran, which reported the highest (78.4%) proportion of primary infertility. This difference may be related to the causes of infertility. Because, in developing countries, STI, abortion, puerperal sepsis, and pelvic inflammatory diseases are common. Such conditions are reported to be a risk factor for secondary infertility and probably be the causes for the higher proportion of secondary infertility [5, 29–31].

In the subgroup analysis, primary infertility was more common in North Africa (70.56%) while secondary infertility was highest in East Africa (69.63%). This finding is also supported by the World health organization (WHO) which reported more prevalent secondary infertility in Sub-Saharan countries [3]. This regional variation may be due to the difference in the etiologic factors. The studies conducted in East Africa reported common risk factors of infertility as; STI, history of abortion and complication during labor, inadequate health service, misuse of antibiotics and antimicrobial resistance [4, 24, 25]. The other subgroup analysis performed by the year of studies showed that the older studies reported a higher pooled proportion of secondary infertility, while recent studies reported a higher pooled proportion of primary infertility. Since older studies might have been conducted when there were poor health care coverage and a high prevalence of infectious causes of infertility, the proportion of secondary infertility is expectedly higher [3, 4].

With regard to the etiologic sources of infertility, female-related causes account for about 54% of the total infertility. This result is in agreement with a meta-analysis report of Agarwal et al. [37], and Eldib and Tashani [38]. Both meta-analyses reported the causes for 50% of infertility were due to female-related reasons, suggesting for priority should be given for the management of risk factors of female origin. Appropriate management of the causes of infertility, since many of them are infections of the reproductive tract or abortion following the first pregnancy, can greatly reduce the prevalence of infertility [30, 31]. However, the burden of all infertility should not be given for women, since 22% of the infertility causes are originated from the male.

In the current meta-analysis, the commonest reported causes of male infertility, oligospermia, and asthenozoospermia are related to the quality of sperm cells. As a result, providing infertile male with treatment that improves sperm quality appear to be important. A meta-analysis on randomized control trial studies reported enhanced sperm quality with supplementation of selenium and coenzyme Q10 [39].

The commonest causes of female infertility were pelvic inflammatory diseases, tubal factors, abortion, and ovulatory dysfunction. Similarly, a meta-analysis by Direkvand-Moghadam et al. reported these factors as causes of female infertility [40]. This indicates that the management of infections affecting the reproductive organs and abortion requires attention.

Overall, infertility is not only a personal issue rather a matter of generation. Therefore, health policymakers and the governments should focus on the provision and advancement of infertility clinics and prevention and management of reproductive tract infection and abortion. Unexplained causes of infertility were also reported, this signals to advance our diagnostic modalities. Some studies were conducted based on institutional diagnostic criteria which made the meta-analysis difficult. Therefore, it is recommended that researches should be conducted following the accepted definition of infertility and diagnostic cut-off points for the assessment of sperm quality. Infertility perplexes the life of especially women, this could be at least vanquished via psychological support.

In conclusion, the current meta-analysis identified an approximately equal proportion of primary and secondary infertility. North Africa and East Africa had more primary and secondary infertility respectively. Older and recent studies respectively reported a higher pooled proportion of secondary and primary infertility. Female related causes were responsible for more than half of infertility. Oligospermia, varicocele, and asthenozoospermia were the commonest reported causes of male infertility. Female infertility was commonly caused by tubal factors, abortion, pelvic inflammatory diseases, and ovulatory dysfunction. The currently available data appears to be not of good quality. This is a call for performing an informative and representative investigation at the level of Africa. On the other hand, interpretation and utilization of these findings should consider the presence of substantial heterogeneity between the included studies.

The current study provides valuable continental data on infertility and its causes, which is useful for regional health policymakers, although has limitations. One of such limitation is it only includes articles in the English language. Since there are many French speaking countries in Africa, it may miss important articles. In addition, some of the articles have a small sample size that may question their representativeness.

Not applicable.

## Data Availability

Further data sets can be accessed from the corresponding author upon request.

## Authors’ information

MS: Lecturer, Department of Anatomy, School of Medicine, College of Health Science, Wollo University, Dessie, Ethiopia.

MA: Associate Professor, Department of Anatomy, College of Health Science, Addis Ababa University, Addis Ababa, Ethiopia.

YA: Lecturer, Department of Biostatistics and Epidemiology, School of Public Health, College of Health Science, Wollo University, Dessie, Ethiopia.
